# Characteristics and outcomes of trauma patients with ICU lengths of stay 30 days and greater: a seven-year retrospective study

**DOI:** 10.1186/cc8054

**Published:** 2009-09-24

**Authors:** Adrian W Ong, Laurel A Omert, Diane Vido, Brian M Goodman, Jack Protetch, Aurelio Rodriguez, Elan Jeremitsky

**Affiliations:** 1 Department of Surgery, Allegheny General Hospital, 320 East North Avenue, Pittsburgh PA 15212, USA; 2 Northfield Laboratories Inc., 1560, Sherman Avenue, Evanston, IL 60201, USA; 3 Department of Cardiology, Allegheny General Hospital, 320 East North Avenue, Pittsburgh PA 15212, USA

## Abstract

**Introduction:**

Prolonged intensive care unit lengths of stay (ICU LOS) for critical illness can have acceptable mortality rates and quality of life despite significant costs. Only a few studies have specifically addressed prolonged ICU LOS after trauma. Our goals were to examine characteristics and outcomes of trauma patients with LOS ≥ 30 days, predictors of prolonged stay and mortality.

**Methods:**

All trauma ICU admissions over a seven-year period in a level 1 trauma center were analyzed. Admission characteristics, pre-existing conditions and acquired complications in the ICU were recorded. Logistic regression was used to identify independent predictors of prolonged LOS and predictors of mortality among those with prolonged LOS after univariate analyses.

**Results:**

Of 4920 ICU admissions, 205 (4%) had ICU LOS >30 days. These patients were older and more severely injured. Age and injury severity score (ISS) were associated with prolonged LOS. After logistic regression analysis, sepsis, acute respiratory distress syndrome, and several infectious complications were important independent predictors of prolonged LOS. Within the group with ICU LOS >30 days, predictors of mortality were age, pre-existing renal disease as well as the development of renal failure requiring dialysis. Overall mortality was 12%.

**Conclusions:**

The majority of patients with ICU LOS ≥ 30 days will survive their hospitalization. Infectious and pulmonary complications were predictors of prolonged stay. Further efforts targeting prevention of these complications are warranted.

## Introduction

Prolonged intensive care unit (ICU) stays for critical illness can result in acceptable mortality rates and quality of life despite significant costs [1,2]. Only a few studies have specifically addressed prolonged ICU lengths of stay (LOS) after trauma [3-5]. Our goals were to determine the outcomes and characteristics of trauma patients with prolonged ICU LOS. Based on previous studies of medical and surgical ICU patients, our hypotheses were that age and injury severity predicted prolonged ICU LOS in trauma patients admitted to the ICU, but that the majority of trauma patients who survived beyond 30 days in the ICU would survive to discharge.

## Materials and methods

This was a retrospective study based on the hospital trauma registry over a seven-year period (1998 to 2004) approved by the hospital Institutional Review Board with waiver of consent. In this level I trauma center, critical care services for injured patients are provided by the same trauma physician group that admits injured patients. Admission clinical characteristics, pre-existing conditions and acquired complications in the ICU were extracted from registry data. Selected definitions used for this study for pre-existing conditions and complications are based on those set by the Pennsylvania Trauma Systems Foundation [see Additional data file 1].

**Figure 1:**
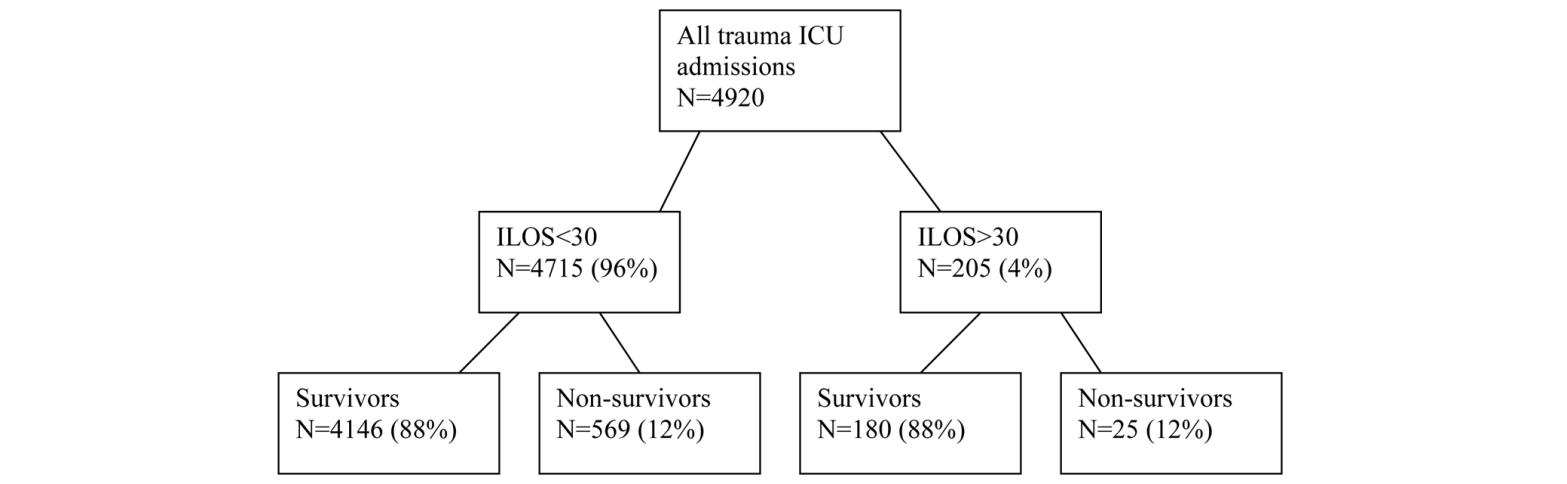
Composition of the study groups. ILOS<30 = patients with intensive care unit (ICU) length of stay less than 30 days; ILOS>30 = patients with ICU length of stay greater than or equal to 30 days.

For the purposes of this study, the control group was designated as those patients who were admitted to the ICU for less than 30 days (ILOS<30). This group was compared with the group with ICU LOS of 30 days or greater (ILOS>30). Within the ILOS>30 group, we also compared survivors with non-survivors (Figure 1).

Data were summarized as mean ± standard deviation. To compare means, we used the independent samples *t *test and the Mann-Whitney U rank sum test. Logistic regression was used to identify independent predictors of prolonged LOS in the entire sample as well as independent predictors of mortality within the ILOS>30 subgroup. Correlation was assessed using Spearman's rho. Chi-squares and nested chi-squares analyses were used to explore relations between variables. Differences were considered significant at *P *< 0.05. SPSS version 14.0 (SPSS Inc., Chicago, IL, USA) was used to analyze the data.

## Results

### Comparison of ILOS>30 and ILOS<30 groups

**Figure 2:**
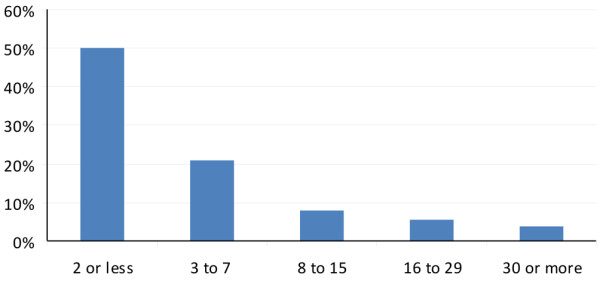
Distribution of length of stay of all trauma ICU patients in the study period. X axis = length of stay (days); Y axis = percentage of all trauma intensive care unit (ICU) patients.

There were 11,035 admissions to the trauma service in the seven-year study period, with 4920 (44.5%) patients admitted to the ICU. ICU LOS for the 4920 patients is shown in Figure 2. The ILOS>30 group (n = 205) had a mean LOS of 45.5 ± 23.8 days (median 39, range 30 to 279 days) with a mean mechanical ventilation duration of 39.9 ± 21.1 days (median 38, range 7 to 192 days). ILOS>30 patients comprised only 4% of all ICU patients, but accounted for 8350 bed days (29%) out of a total of 28,771 bed days and 6742 ventilator days (41%) out of a total of 16,335 during this study period.

Demographic and clinical characteristics are shown in Table 1. ILOS>30 patients were significantly older, more severely injured, and had lower Glasgow Coma Scores (GCS) on admission. A modest positive correlation existed between illness severity score (ISS) and ICU LOS (Spearman's rho = 0.4, *P *< 0.001). The LOS>30 patients group also had significantly higher incidences of pre-existing cardiac, renal, pulmonary conditions and diabetes mellitus. Not surprisingly, ILOS>30 patients sustained significantly more complications in the ICU.

**Table 1:** Demographic and clinical characteristics for ILOS>30 and ILOS<30 groups

Characteristic	ILOS < 30 (n = 4716)	ILOS > 30 (n = 205)	*P *value
Age (in years)	48.3 ± 23.4 (Median = 45.0)	54.7 ± 21.6 (Median = 55.0)	<0.001*
Age ≥ 65 years (%)	1415/4716 (30)	80/205 (39)	0.006*
Gender (% male)	65.8	72.2	0.07
Mechanism of injury (% blunt)	93.7	95.1	0.5
Injury severity score	18.0 ± 11.2 (Median = 17.0)	28.4 ± 13.1 (Median = 26.0)	<0.001*
Glasgow coma score	12.3 ± 4.6 (Median = 15.0)	10.6 ± 5.3 (Median = 14.0)	<0.001*
**Pre-existing conditions (%)**			
Dialysis	0.5	0.5	1.0
Cardiac	28.7	38.5	0.003*
Renal	0.8	2.4	0.049*
Pulmonary	7.4	13.2	0.003*
Neurological	9.0	6.8	0.4
Liver	1.1	0.5	0.6
Warfarin use	4.4	3.4	0.6
Diabetes	10.1	18.0	<0.001*
Immunological	0.2	0.5	0.8
Drug abuse	5.5	4.9	0.8
Alcohol abuse	9.4	11.2	0.5
Psychological	10.3	8.8	0.6
**Complications (%)**			
Arrest	0.8	7.3	<0.001*
Pneumonia	5.2	54.1	<0.001*
Sepsis	1.7	31.2	<0.001*
Urinary tract infection	6.8	42.0	<0.001*
Deep vein thrombosis	1.4	10.2	<0.001*
Pulmonary embolus	0.6	2.9	<0.001*
Myocardial infarction	1.1	5.4	<0.001*
Arrhythmia	3.5	25.9	<0.001*
Empyema	0.1	2.9	<0.001*
Acute respiratory distress syndrome	2.1	43.4	<0.001*
Renal	1.5	20.0	<0.001*
Gastrointestinal bleed	0.7	9.3	<0.001*
Liver	0.2	2.4	<0.001*
Respiratory	6.9	45.9	<0.001*
Dialysis	0.3	4.9	<0.001*
**Mortality rate (%)**	12.1	12.2	1.0

*statistically significant.

ILOS<30 = patients with intensive care unit length of stay less than 30 days; ILOS>30 = patients with intensive care unit length of stay greater or equal to 30 days.

Of the 4920 patients, 3421 (69.5%) were younger than 65 years old compared with 1499 (30.5%) who were 65 years old or older. ICU LOS was significantly associated with patient age (<65 versus >65 years old) when controlled for injury severity except in the least severely injured and most severely injured categories (Table 2). Age was also significantly associated with mortality. Patients 65 years and older had a mortality rate of 24.4% compared with 6.7% for younger patients (*P *< 0.001). When controlled for injury severity, the association of mortality with age was significant for all degrees of injury severity (Table 3). For the ISS 1-3 patients who died, three had no autopsies (and therefore potential injuries may not have been delineated completely), three suffered anoxic brain injury after hanging and drowning accidents, and one died from necrotizing fasciitis after sustaining minor soft tissue trauma 10 days previously.

**Table 2:** Relation between age and intensive care unit length of stay

ISS category	ILOS<30 (%)	ILOS>30 (%)	Total patients	*P*
ISS 1-3 Age <65 years	113	1 (0.8)	114	0.4
Age ≥ 65 years	25	0 (0)	25	
				
ISS 4-8 Age <65 years	491	0 (0)	491	0.009*
Age ≥ 65 years	131	3 (2.3)	134	
				
ISS 9-15 Age <65 years	907	10 (1.1)	917	<0.001*
Age ≥ 65 years	352	17 (4.8)	369	
				
ISS 16-24 Age <65 years	876	19 (2.2)	895	0.003*
Age ≥ 65 years	450	25 (5.6)	475	
				
ISS 25-75 Age <65 years	899	94 (10.5)	993	0.2
Age ≥ 65 years	457	36 (7.9)	493	

* statistically significant.

ILOS<30 = patients with intensive care unit length of stay less than 30 days; ILOS>30 = patients with intensive care unit length of stay greater or equal to 30 days; ISS = injury severity score.

**Table 3:** Relation between age and mortality

ISS category	Mortality rate (%)	*P *value
ISS 1-3 Age <65 years	3/114 (2.6)	0.02*
Age ≥ 65 years	4/25 (16)	
		
ISS 4-8 Age <65 years	1/491 (0.2)	<0.001*
Age ≥ 65 years	15/134 (11.1)	
		
ISS 9-15 Age <65 years	12/917 (1.3)	<0.001*
Age ≥ 65 years	44/369(11.9)	
		
ISS 16-24 Age <65 years	19/895(2.1)	<0.001*
Age ≥ 65 years	69/475(14.5)	
		
ISS 25-75 Age <65 years	194/993(19.5)	<0.001*
Age ≥ 65 years	233/493(47.3)	

*statistically significant. ISS = injury severity score.

Univariate analysis produced the following predictors of ICU stay of more than 30 days: age over 65 years, ISS > 21 (Receiver operating characterstic curve [ROC] analysis; sensitivity 72% [95% C.I. 65%, 78%], specificity 64% [95% C.I. 63%, 66%]), GCS <12 (ROC curve analysis; sensitivity 43% [95% C.I. 36%, 50%], specificity 73% [95% C.I. 72%, 75%]), pre-existing cardiac, renal, pulmonary or diabetic conditions, and complications that developed during ICU stay (all *P *< 0.05).

Variables with *P *< 0.2 by univariate analysis were entered into a logistic regression analysis to create a prediction model for ICU LOS of 30 days or longer. The *P *value was set at 0.2 because some variables may prove to have lower *P *values in a model or to be important confounders. Further, many variables in this set were of special interest to us because they had been found previously to be important predictors.

Male gender, ISS, or the presence of cardiopulmonary arrest, pneumonia, acute respiratory distress syndrome (ARDS), respiratory failure requiring intubation or re-intubation, urinary tract infection, deep vein thrombosis, arrhythmias, sepsis, or gastrointestinal bleed were found to be independent predictors of LOS of more than 30 days (Table 4). The occurrences of sepsis and ARDS, in particular, increased the odds by 5.0 and 8.8, respectively, of prolonging ICU stay of more than 30 days. This model correctly predicted 96% of outcomes. An increase in the ISS of 1 resulted in a 4% increase in the odds of ICU LOS >30 days.

**Table 4:** Independent predictors of intensive care unit length of stay of 30 days or longer by logistic regression analysis

Parameter	β	SE	*P *value	Exp (β)
Constant	-5.4	0.37	<0.001	0.004
Male gender	-0.53	0.22	0.02	0.59
Injury severity score	0.04	0.008	<0.001	1.04
Pneumonia	1.58	0.20	<0.001	4.87
Arrest	1.35	0.47	0.004	3.87
Sepsis	1.80	0.26	<0.001	6.02
Urinary tract infection	1.56	0.22	<0.001	4.75
Deep vein thrombosis	1.10	0.37	0.003	2.99
Arrhythmias	0.90	0.26	0.001	2.45
Acute respiratory distress syndrome	2.29	0.23	<0.001	9.83
				
Gastrointestinal bleed	0.93	0.42	0.03	2.54
Respiratory	1.51	0.20	<0.001	4.53

Cox-Snell R square = 0.16

Nagelkerke R square = 0.54.

β = logistic coefficient (parameter estimate); Exp (β) = odds ratio; SE = standard error of logistic coefficient.

### ILOS>30 group: survivors versus non-survivors

Within the ILOS>30 group, non-survivors were significantly older and had longer durations of mechanical ventilation (Table 5). ISS and GCS on admission were similar. Univariate analysis showed that besides age and female gender, death was significantly associated with pre-existing cardiac, renal and neurological conditions, and the following complications: myocardial infarction, arrhythmias, renal failure, ARDS and the requirement for renal replacement therapy.

**Table 5:** Characteristics of the group of patients with intensive care unit length of stay more than 30 days by survival status

Characteristic	Survived (n = 180)	Died (n = 25)	*P *value
Age (in years)	52.5 ± 21.6 (Median = 53.0)	70.3 ± 13.5 (Median = 73.0)	<0.001*
Age ≥ 65 years (%)	62/180 (34)	18/25 (72)	<0.001*
Gender (% male)	75	52	0.03*
Mechanism (% blunt)	94.4	100	0.5
Intensity severity score	28.9 ± 13.2	24.5 ± 12.1	0.1
Glasgow coma score	10.3 ± 5.4 (Median = 14.0)	12.5 ± 4.2 (Median = 15.0)	0.2
Ventilator days	38.1 ± 17.0 (Median = 36.0)	53.3 ± 37.7 (Median = 40.0)	0.03*
**Pre-existing conditions (%)**			
Dialysis	0.6	0	1.0
Cardiac	34.4	68.0	0.003*
Renal	1.1	12.0	0.009*
Pulmonary	13.3	12.0	1.0
Neurological	5.0	20.0	0.02*
Liver	0.6	0	1.0
Warfarin use	2.2	12.0	0.053
Diabetes	17.2	24.0	0.6
Immunological	0.6	0	1.0
Drug abuse	5.0	4.0	1.0
Alcohol abuse	12.2	4.0	0.4
Psychological	8.9	8.0	1.0
**Complications (%)**			
Arrest	6.7	12.0	0.6
Pneumonia	53.9	56.0	1.0
Sepsis	33.3	16.0	0.1
Urinary tract infection	41.1	48.0	0.7
Deep vein thrombosis	10.0	12.0	1.0
Pulmonary embolus	2.8	4.0	1.0
Myocardial infarction	3.9	16.0	0.04*
Arrhythmia	23.3	44.0	0.049*
Empyema	0.1	0.4	0.4
Acute respiratory distress syndrome	1.6	5.6	<0.001*
Renal	0.8	7.0	<0.001*
Gastrointestinal bleed	8.3	16	0.3
Liver	2.2	4	0.5
Respiratory	43.3	64	0.06
Dialysis	3.3	16	0.02*

* statistically significant

After variables with *P *< 0.2 by univariate analysis were entered into a logistic regression analysis, age, pre-existing renal conditions and need for renal replacement therapy emerged as independent predictors of death in the ILOS>30 group. The odds of death increased by 4.7 and 9.1, respectively, if there was a need for dialysis and if there was a pre-existing renal condition. With every year of age, the odds of death increased by 5%. This model correctly predicted outcomes in 88% of patients. Cause of death was multiple-organ failure (MOF) in 22 patients, acute respiratory failure in two patients and sudden massive hemoptysis due to necrotizing *Mycobacterium *pneumonia in one. Overall mortality rate in the ILOS>30 group was 12%.

### Discharge destinations for survivors

Sixty-one percent of patients with ICU LOS of less than 30 days were discharged home as compared with 8% of patients with ICU LOS of 30 days or more (*P *< 0.001; Table 6). The majority of the ILOS>30 survivors were transferred to inpatient rehabilitation centers (55%) and skilled nursing facilities (28%).

**Table 6:** Discharge destinations for survivors (ILOS<30 versus ILOS>30)

Discharge destination (%)	ILOS<30 (n = 4106)	ILOS>30 (n = 180)	Total (n = 4286)
Home	61.2	7.8	58.9
Inpatient rehabilitation	22.1	54.4	23.4
Skilled nursing facility	11.0	28.3	11.8
Long-term acute care facility	0	2.8	0.2
Other (Burn center, psychiatric facility etc.)	5.7	6.7	5.7

ILOS<30 = patients with intensive care unit (ICU) length of stay less than 30 days; ILOS>30 = patients with ICU length of stay greater than or equal to 30 days.

## Discussion

Only a few studies have specifically addressed prolonged ICU stays in trauma patients. Trottier and colleagues [3] analyzed 339 trauma and burn patients with ICU LOS of more than 28 days and found similar survival rates (87%) to our study with age being the most important predictor of outcome. Compared with a control group of patients with shorter LOS, the authors demonstrated that age, injury severity, and the presence of burn injuries were determinants of prolonged ICU stay. Miller and colleagues [4] found that the overall mortality rate was 22% with the majority of patients dying from MOF. Age was the only significant predictor of mortality. In both these studies, pre-existing conditions were not analyzed. Goins and colleagues [5] reported a mortality rate of 17% for 87 trauma patients spending more than 30 days in the ICU. There was no comparison to a control group.

In contrast to the above-mentioned studies, our study was unique in that we analyzed differences in pre-existing conditions and acquired complications. We found that ILOS>30 patients constituted only a small percentage of all trauma admissions to the ICU but consumed a disproportionately large amount of ICU resources. These findings are similar to a prospective study by Martin and colleagues where in a heterogeneous population, prolonged-stay patients represented 5.6% of ICU admissions and accounted for almost 40% of bed days [6]. Similarly, medical-surgical ICU patients with ICU LOS of more than 30 days accounted for 8% of total ICU admissions but 48% of occupied beds [7] in another study.

Not surprisingly, age and injury severity were associated with prolonged ICU stay and mortality, but after multivariate analysis, age was not found to be an independent predictor of prolonged stay, and neither were pre-existing conditions or admission GCS. Instead, sepsis, ARDS and other infectious complications were found to be powerful predictors.

That age or pre-existing conditions did not independently predict prolonged stay could simply be attributed to selection bias: older patients and those with significant pre-existing conditions may not have survived to the 30-day mark. This is suggested by comparing those who died before 30 days to the ILOS>30 patients: patients who died before 30 days of admission were older, and more likely to have a significant head injury, pre-existing cardiac or neurological condition and be on warfarin. Notably, in ILOS<30 non-survivors, 61% were aged 65 years or older versus 39% in the ILOS>30 group.

Within the ILOS>30 group, similar to the previous studies on trauma patients, we found that age was still an independent predictor of mortality. In addition, pre-existing renal conditions and the need for renal replacement therapy during the ICU stay also predicted mortality. The high mortality rates associated with dialysis have been reported in other institutions [2,7,8]. The study by Eachempati and colleagues [8] demonstrated a mortality rate of 61% in patients requiring dialysis compared with an overall mortality of 45% for all patients with acute renal failure. Patients who required dialysis in our study had a mortality rate of 33%.

The mortality rate in the ILOS>30 trauma patients (12%) was consistent with the previously published studies on trauma patients. This finding could be used to support families who may be discouraged by the length of time their family member is in the ICU, as well as to illustrate to health care providers that their efforts are not in vain. In a prospective observational study [9], there were discordant predictions with regard to futility of survival and quality of life between doctors and nurses in 21% of ICU patients. Only 9 to 15% of survivors of ICU stay where health care professionals had considered treatment futile actually reported bad quality of life six months later. On the other hand, physician estimates of ICU survival can be powerful predictors of ICU mortality when compared with illness severity, organ dysfunction and the use of inotropic drugs, possibly by contributing to more 'do not resuscitate' directives in instances of cardiac arrest, and more likely withdrawal of dialysis, pharmacological support, and mechanical ventilation [10].

That patients aged 65 years and older accounted for almost 40% of the ILOS>30 group was reflective of our admission population, where these elderly patients comprised 28% of all trauma admissions to our institution. Older trauma patients have been recognized as having a higher risk of dying when chronic medical conditions exist compared with those without chronic conditions, and this relation between mortality and pre-existing medical conditions is more apparent when these patients sustain less severe injuries [11]. Studies in non-trauma ICU cohorts support the conclusion that age in and of itself does not predict poor outcome [12-14]. Higgins and colleagues [14] determined that the need for ventilation at 24 hours, trauma and emergency surgery admissions, severity of illness, and prolonged pre-ICU stays were independent predictors of prolonged stay, and not age in itself. Pre-hospital functional status has also been found to be an important predictor of poor outcome in ICU patients [15-17].

There were several limitations of this study. One was the lack of data on long-term outcome and pre-injury functional status. We also did not have prospective information on prognostic indicators of ICU survivability or measures of organ dysfunction with time in the ICU. Also, we could not assess the degree of adherence to evidence-based practices known to reduce ICU morbidity and mortality such as glycemic control, sedation protocols, ventilator practices, and transfusion and phlebotomy practices [18]. Further, ICU LOS was influenced to a certain extent by discharge planning arrangements with insurance payers and transfer facilities. The lack of prospective time-dependent data regarding organ dysfunction and the degree of adherence to evidence-based guidelines makes it difficult to determine to what extent the acquired ICU complications were a result of sub-optimal ICU care rather than nature of disease due to the injuries sustained on admission.

Finally, the definitions of certain pre-existing conditions such as cardiac and pulmonary disease lacked objective criteria. This was because these criteria were frequently not available for trauma patients admitted as emergencies to the ICU. As these cardiac and pulmonary conditions were factors that were entered into the logistic regression analysis (Table 4), it is conceivable that were the definitions modified by including objective criteria, they could have emerged as independent risk factors predicting prolonged ICU stay.

## Conclusions

Trauma patients who spent 30 days or more in the ICU consumed a disproportionate amount of ICU resources. For those who survived to 30 days, acquired pulmonary and infectious complications were important predictors of prolonged stay. Although injury severity was found to be an independent predictor of ICU LOS of 30 days or more, partly confirming our hypothesis, age was not. Age, however, did predict mortality in the patients with LOS of 30 days or more, together with pre-existing renal disease and the development of renal failure in the ICU requiring renal replacement therapy. The majority (88%) of these prolonged-stay patients also survived to discharge, confirming our second hypothesis. These findings imply that resources should continue to be directed at infection prevention and surveillance in trauma ICU patients, and also underscores the necessity of adhering to evidence-based guidelines that may decrease ICU LOS. We feel that this study suggests associations between variables in a broad spectrum of trauma patients and communicates important trauma outcomes, and that the data could provide a framework for the generation of hypotheses about prolonged ICU stay in a trauma patient population.

## Key messages

• Trauma patients who have ICU LOS of 30 days or more constituted only 4% of all trauma ICU admissions but accounted for a disproportionate usage of ICU resources.

• 88% of these patients survived to hospital discharge.

• Infectious complications, sepsis, ARDS were independent predictive factors for ICU LOS of 30 days or more.

• Mortality in these prolonged-stay patients was influenced by age, development of renal failure requiring renal replacement therapy, and pre-existing renal dysfunction.

## Abbreviations

ARDS: acute respiratory distress syndrome; GCS: Glasgow Coma Score; ICU: intensive care unit; ILOS<30: patients with ICU length of stay less than 30 days; ILOS>30: patients with ICU length of stay greater or equal to 30 days; ISS: Injury Severity Score; LOS: length of stay; MOF: multiple organ failure.

## Competing interests

The authors declare that they have no competing interests.

## Authors' contributions

AO analyzed the data, participated in the study design and drafted the manuscript. LO conceived of the study, participated in the study design and helped draft the manuscript. DV participated in the study design and analyzed the data, and helped draft the manuscript. BG collected the data, participated in the study design and analysis of the data. JP collected the data, participated in the study design, and helped draft the manuscript. EJ conceived of the study with LO, collected the data and participated in the study design. AR participated in the study design and helped draft the manuscript. All authors read and approved the final manuscript.

## Supplementary Material

Additional file 1 A Word file containing a list of selected definitions used in this study. This is a list of definitions of selected complications and pre-existing conditions based on the Pennsylvania Trauma Systems Foundation 2008 Operations Manual for the Pennsylvania Data Base Collection System. Click here for file
