# Supplementary material for: Characteristics and outcomes of trauma patients with ICU lengths of stay 30 days and greater: a seven-year retrospective study
Source: Crit Care. 2009 Sep 24;13(5):R154. doi: 10.1186/cc8054 (PMC2784377; doi:10.1186/cc8054)
Supplement: Additional file 1 — A Word file containing a list of selected definitions used in this study. This is a list of definitions of selected complications and pre-existing conditions based on the Pennsylvania Trauma Systems Foundation 2008 Operations Manual for the Pennsylvania Data Base Collection System. [file cc8054-S1.DOC]

**Selected definitions used in this study**

# Complications:

Arrest: cardiopulmonary arrest not resulting in death

Arrhythmia: dysrhythmia requiring drugs or defibrillation (not resulting in death)

Respiratory: need for prolonged ventilatory support after a period of normal non-assisted breathing (minimum of 48 hours) or reintubation

Renal: defined by one of the following—(1) creatinine >3.5, (2) BUN>100 mg/dl, (3) development of anuria (4) patient receiving acute renal dialysis to reverse their current renal failure

Gastrointestinal bleed: blood loss from anywhere in the GI tract, grossly positive gastric aspirate or stool requiring treatment.

Liver: documented by a physician. Increased serum ammonia or decreased synthetic or metabolic function.

**Pre-existing conditions:**

Cardiac: history of cardiac surgery, coronary artery disease, congestive heart failure, cor pulmonale, myocardial infarction, hypertension, congenital cardiac disease.

Renal: serum creatinine >2 mg/dl on admission, dialysis on admission.

Pulmonary: documented prior history with ongoing active treatment, asthma, chronic obstructive pulmonary disease, chronic pulmonary condition

Immunosuppressed: HIV/AIDS, routine steroid therapy, transplants, active chemotherapy

Psychological: Attention Deficit Disorder, Attention Deficit Hyperactivity Disorder, mental retardation.

Liver: Bilirubin >2 ml/dl on admission, documented history of cirrhosis

Neurological: spinal cord injury, multiple sclerosis, Alzheimer’s disease, seizure, chronic demyelinating disease, chronic dementia, organic brain syndrome, Parkinson’s disease, cerebrovascular accident.
